# Supplementary material for: Environment and evolutionary history shape phylogenetic turnover in European tetrapods
Source: Nat Commun. 2019 Jan 16;10:249. doi: 10.1038/s41467-018-08232-4 (PMC6335467; doi:10.1038/s41467-018-08232-4)
Supplement: Supplementary file 1 — Supplementary Information [file 41467_2018_8232_MOESM1_ESM.pdf]

Supplementary Information

Environment and evolutionary history shape phylogenetic turnover in European tetrapods

Saladin et al.

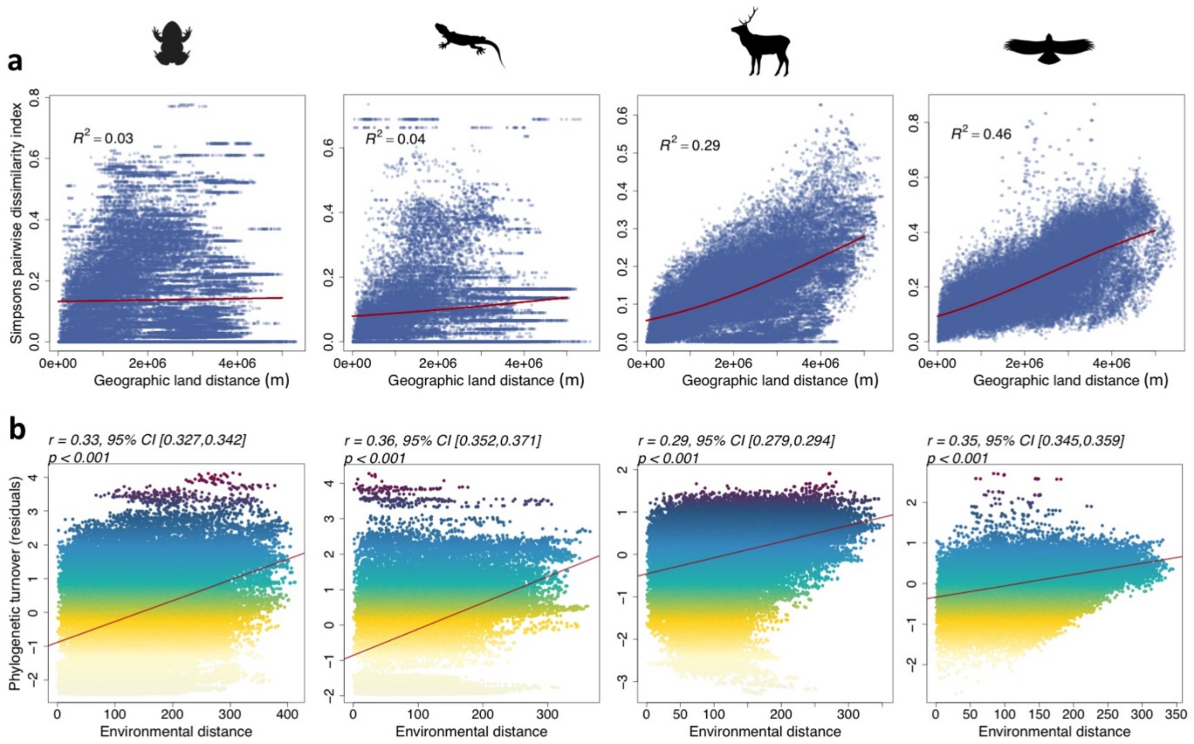

Supplementary Fig 1. Relationship between phylogenetic turnover and geographic and environmental distance in tetrapods. a Relationship between true phylogenetic turnover (Simpsons pairwise dissimilarity index) and geographic land distance among pairs of sample sites for amphibians (1st column), squamates (2nd column), mammals (3rd column) and birds (4th column). R^2 represents the model fit of a logistic regression (Simpsons pairwise dissimilarity index ~ geographic land distance) and the red lines represent the model predictions. **b** Relationship between phylogenetic turnover (after removal of geographic distance effects) and environmental distance. The Pearson correlation (*r*) with 95% Confidence Interval (*CI*) and statistical p-value (*p*) are provided, together with the model prediction from a regression (red line). Silhouette images were taken unchanged from phylopic.org, courtesy of Pedro de Siracusa (amphibians), Ghedo and T. Michael Keesey (squamates) both available under CC BY-SA 3.0 (creativecommons.org/licenses/by-sa/3.0/), Steven Traver (mammals) available under CC0 1.0 (creativecommons.org/publicdomain/zero/1.0), and Shyamal (birds) available under CC BY 3.0 (creativecommons.org/licenses/by/3.0/).

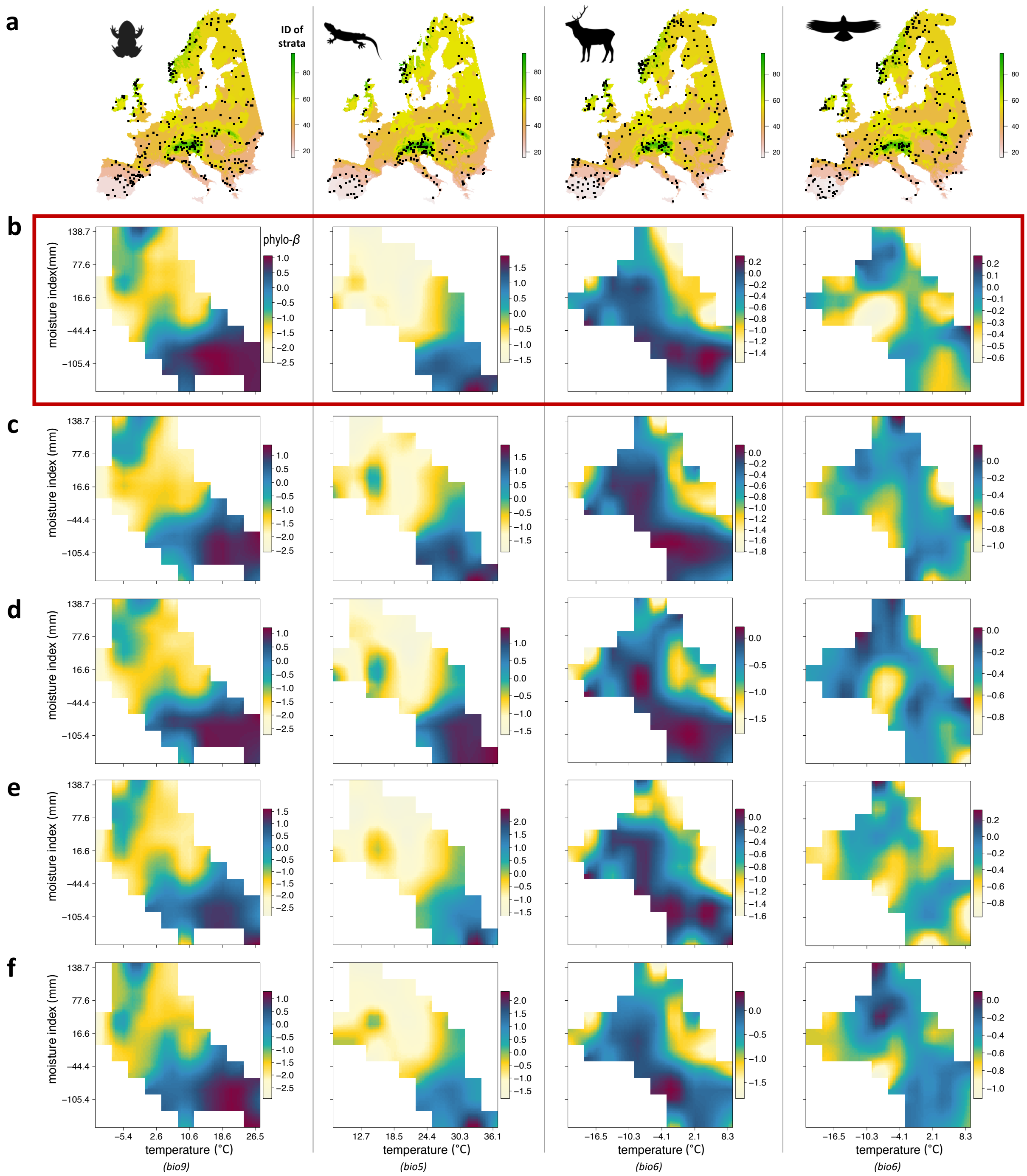

Supplementary Fig 2. Robustness of phylo-*ß* regarding sampling site allocation. a Spatial representation of the first out of five random samplings of communities within each stratum. **b-f** True phylogenetic turnover (phylo-*ß*) in environmental space for amphibians (1st column), squamates (2nd column), mammals (3rd column) and birds (4th column) using five repeated random samples (**b-f**) of communities. Results within the red bar (**b**) represent those used in the main text and are therefore identical to Figure 2. Silhouette images were taken unchanged from phylopic.org, courtesy of Pedro de Siracusa (amphibians), Ghedo and T. Michael Keesey (squamates) both available under CC BY-SA 3.0 (creativecommons.org/licenses/by-sa/3.0/), Steven Traver (mammals) available under CC0 1.0 (creativecommons.org/publicdomain/zero/1.0/), and Shyamal (birds) available under CC BY 3.0 (creativecommons.org/licenses/by/3.0/).

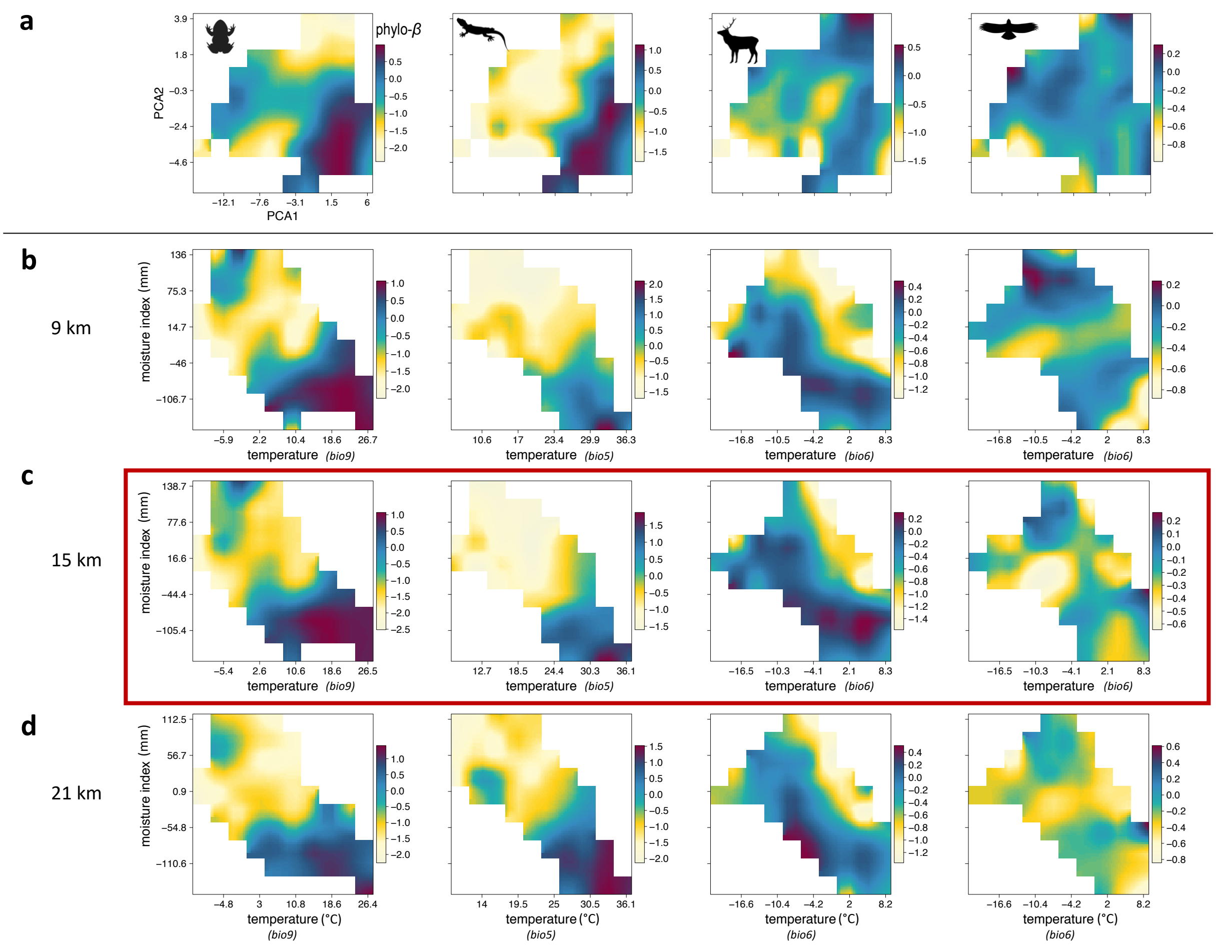

Supplementary Fig. 3. Robustness of phylo-*ß* regarding the representation of climate axes (a) and the spatial resolution of the distribution data (b-d). True phylogenetic turn-over (phylo-*ß*) is represented in environmental space (after removal of geographic distance effects) for amphibians (1st column), squamates (2nd column), mammals (3rd column), and birds (4th column). **a** Phylo-*ß* is mapped along the first two PCA axes instead of two explicit bioclim variables (see Supplementary Table 1 for the correlation of bioclim variables with the PCA axes). **b-d** Phylo-*ß* is calculated and mapped using different spatial resolutions of the distribution data. Results within the red bar (**c**) represent those used in the main text and are therefore identical to Figure 2. Silhouette images were taken unchanged from phylopic.org, courtesy of Pedro de Siracusa (amphibians), Ghedo and T. Michael Keesey (squamates) both available under CC BY-SA 3.0 (creativecommons.org/licenses/by-sa/3.0/), Steven Traver (mammals) available under CC0 1.0 (creativecommons.org/publicdomain/zero/1.0), and Shyamal (birds) available under CC BY 3.0 (creativecommons.org/licenses/by/3.0/).

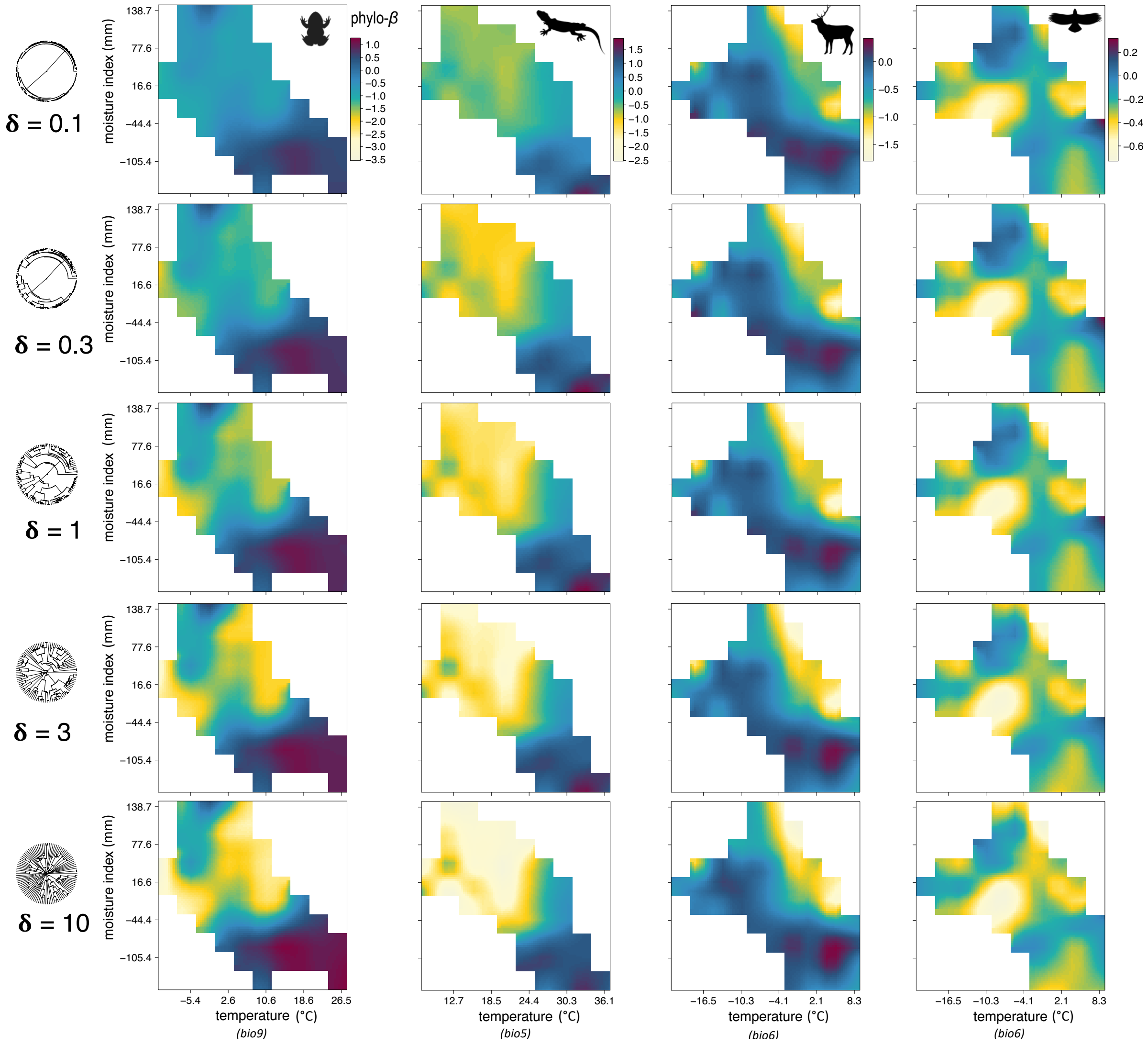

Supplementary Fig. 4. **Effect of *δ*-transformation on phylo-*β* patterns.** True phylogenetic turnover (phylo-*β*) is represented in environmental space (after removal of geographic distance effects) for amphibians (1st row), squamates (2nd row), birds (3rd row), and mammals (4th row) using rescaled phylogenetic trees. Phylo-*β* was calculated by using rescaled phylogenetic trees based on delta transformation ranging from *δ*=0.1 (disproportionately increasing the length of recent branches) to *δ*=10 (disproportionately increasing the length of deep branches). *δ*=1 represents unscaled phylogenetic trees. Silhouette images were taken unchanged from phylopic.org, courtesy of Pedro de Siracusa (amphibians), Ghedo and T. Michael Keesey (squamates) both available under CC BY-SA 3.0 (creativecommons.org/licenses/by-sa/3.0/), Steven Traver (mammals) available under CC0 1.0 (creativecommons.org/publicdomain/zero/1.0), and Shyamal (birds) available under CC BY 3.0 (creativecommons.org/licenses/by/3.0/).

a

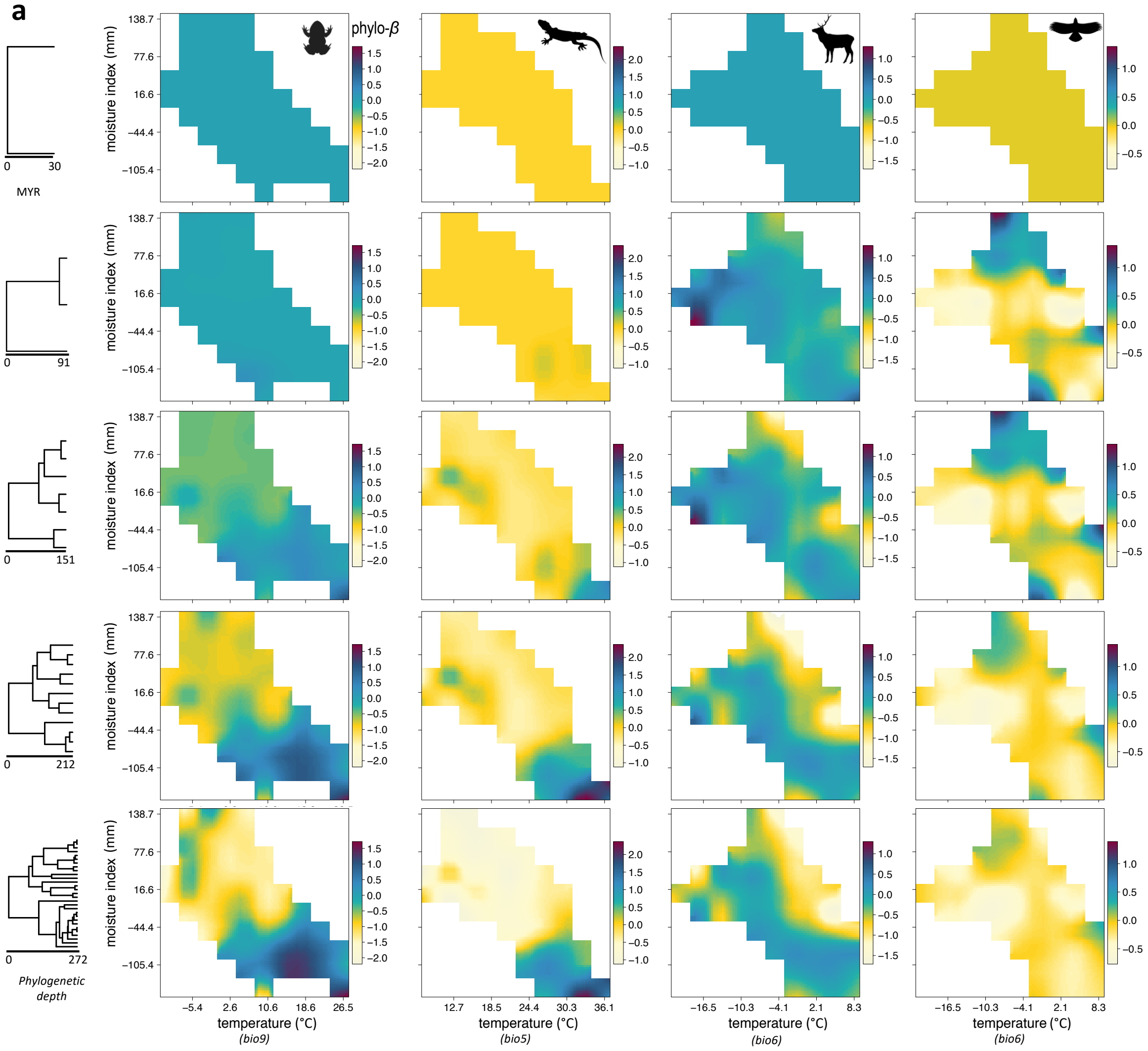

phylo-β
moisture index (mm)
138.7
77.6
16.6
−44.4
−105.4
temperature (°C)
(bio9)
(bio5)
(bio6)
(bio6)
−5.4
2.6
10.6
18.6
26.5
12.7
18.5
24.4
30.3
36.1
−16.5
−10.3
−4.1
2.1
8.3
0
30
MYR
91
151
212
272
Phylogenetic depth

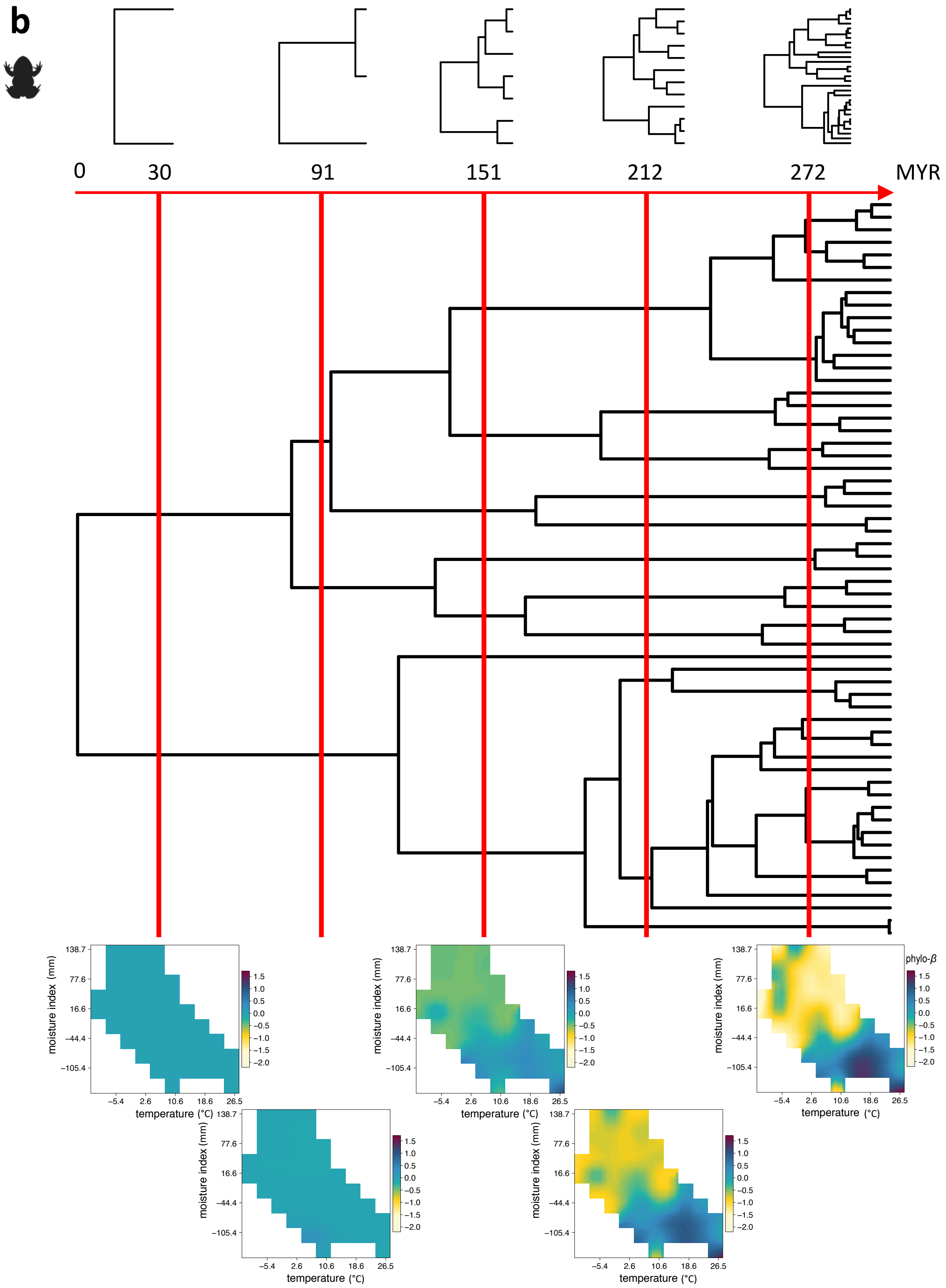

b
0
30
91
151
212
272
MYR
moisture index (mm)
138.7
77.6
16.6
−44.4
−105.4
temperature (°C)
−5.4
2.6
10.6
18.6
26.5
1.5
1.0
0.5
0.0
−0.5
−1.0
−1.5
−2.0
phylo-β

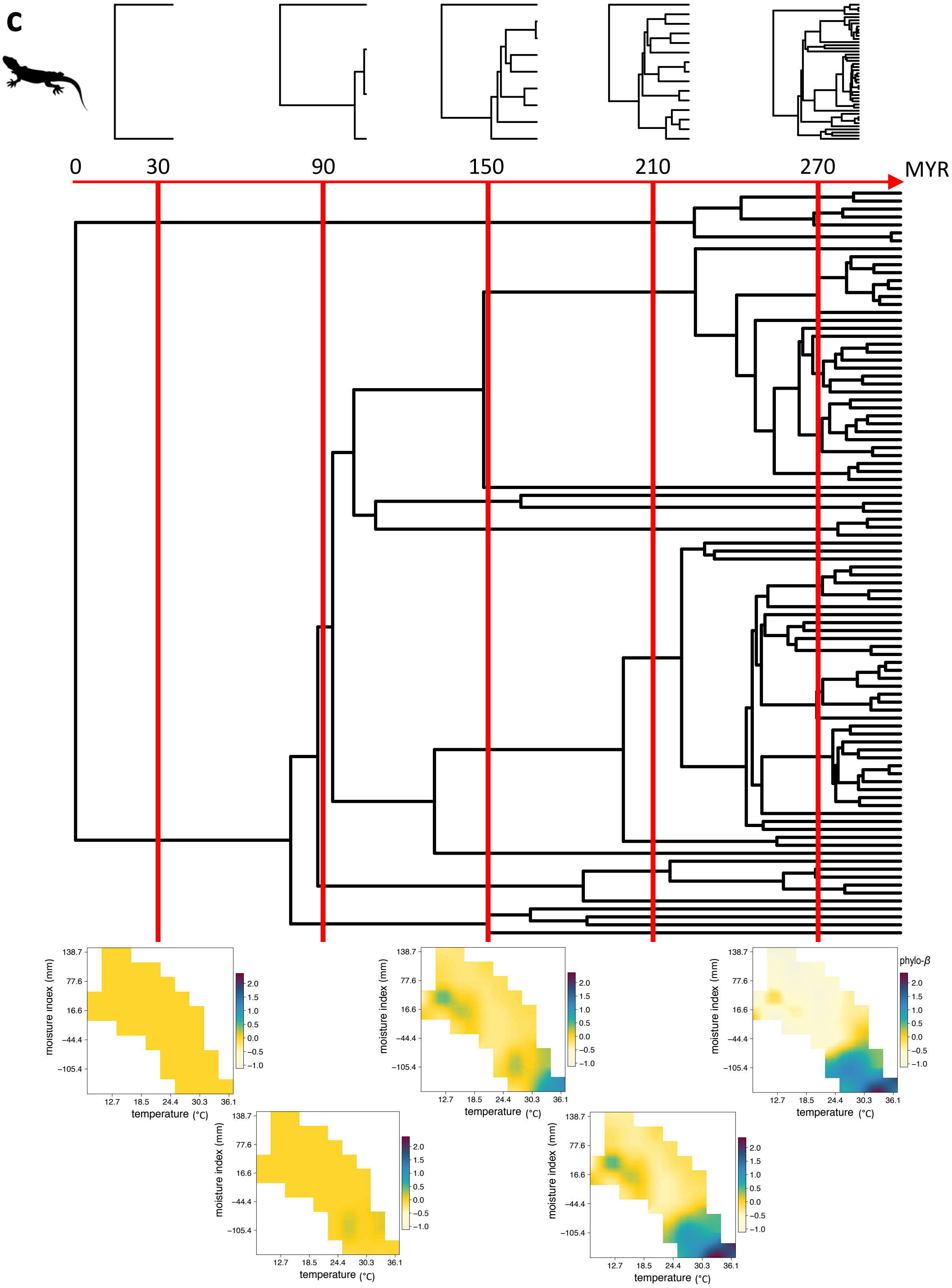
c
0
30
90
150
210
270
MYR
moisture index (mm)
138.7
77.6
16.6
−44.4
−105.4
temperature (°C)
12.7
18.5
24.4
30.3
36.1
phylo-β
2.0
1.5
1.0
0.5
0.0
−0.5
−1.0

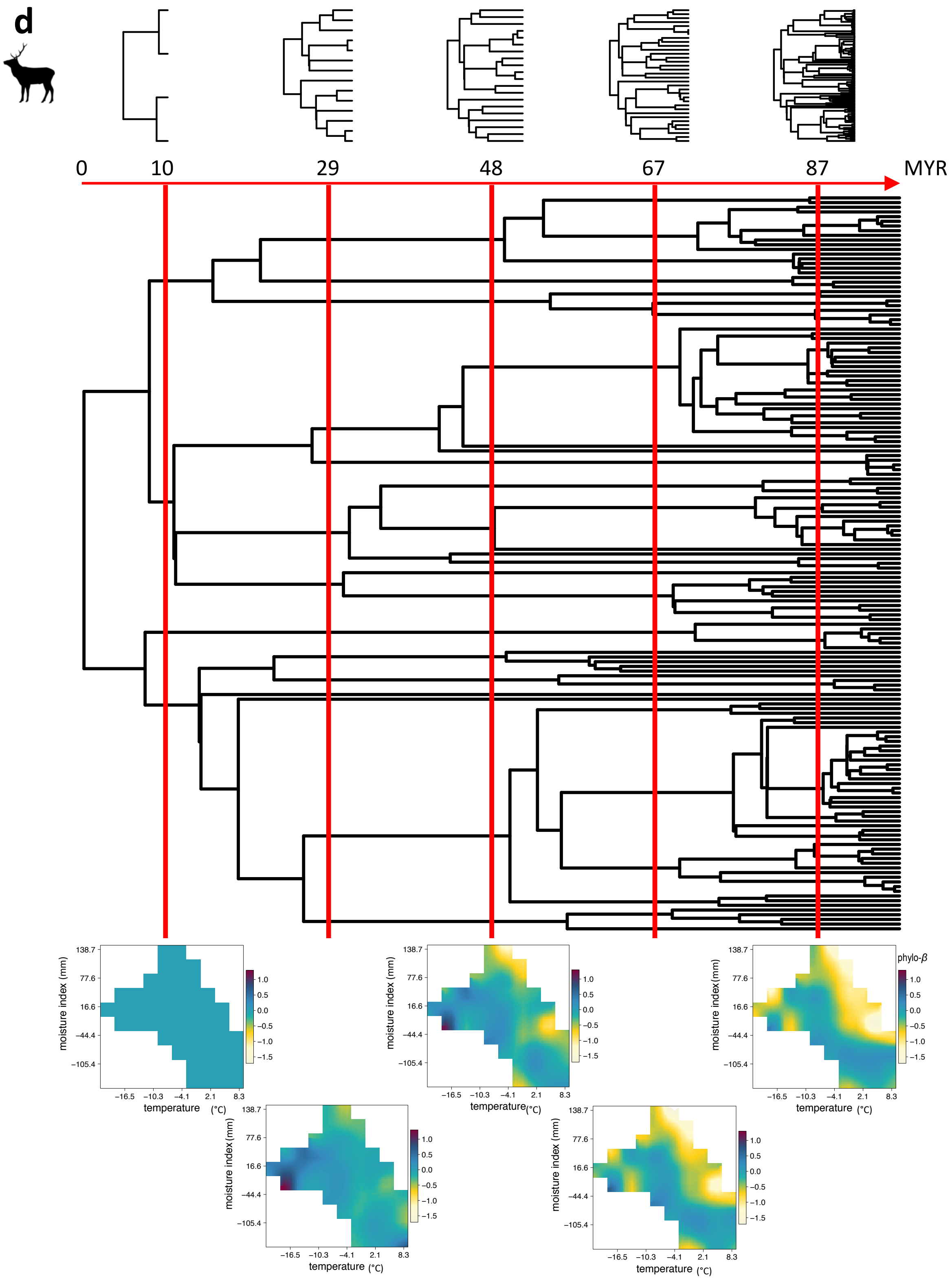
d
0
10
29
48
67
87
MYR
moisture index (mm)
temperature (°C)
phylo-β
138.7
77.6
16.6
−44.4
−105.4
−16.5
−10.3
−4.1
2.1
8.3
1.0
0.5
0.0
−0.5
−1.0
−1.5

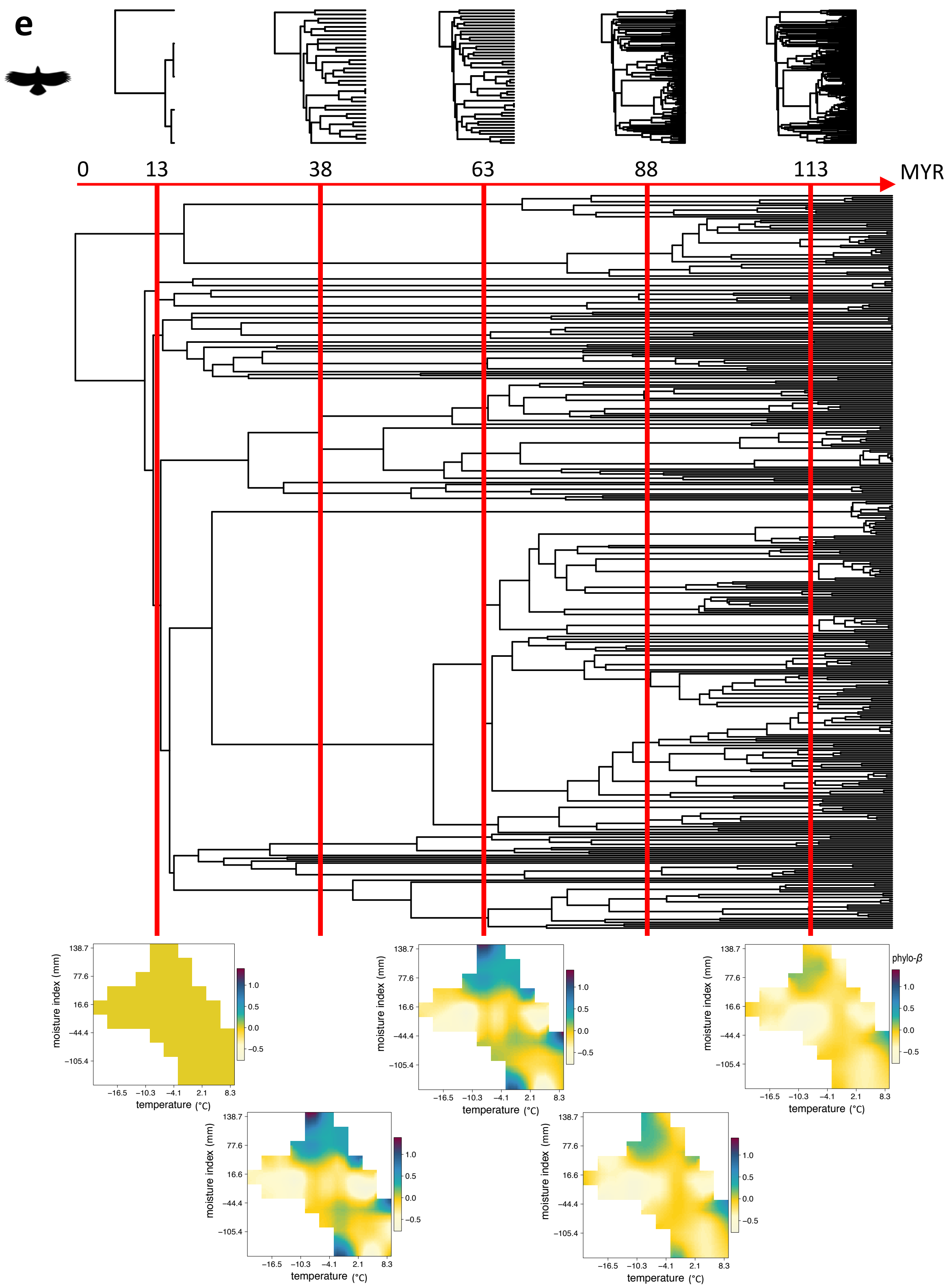

Supplementary Fig. 5. **Effect of tree truncation at different phylogenetic depths on phylogenetic turnover patterns in environmental space. a** True phylogenetic turnover (phylo-β) is represented in environmental space (after removal of geographic distance effects) for amphibians (1st row), squamates (2nd row), birds (3rd row), and mammals (4th row) using truncated phylogenetic trees sliced at different phylogenetic depths (MYR: million years of evolution since clade origin; as illustrated on the left side with the amphibian phylogeny). **b-e** The same results are represented but along the cut levels (MYR of evolution) across the phyloge-

nies for amphibians (**b**), squamates (**c**), mammals (**d**) and birds (**e**). Silhouette images were taken unchanged from phylopic.org, courtesy of Pedro de Siracusa (amphibians), Ghedo and T. Michael Keesey (squamates) both available under CC BY-SA 3.0 (creativecommons.org/licenses/by-sa/3.0/), Steven Traver (mammals) available under CC0 1.0 (creativecommons.org/publicdomain/zero/1.0), and Shyamal (birds) available under CC BY 3.0 (creativecommons.org/licenses/by/3.0/).

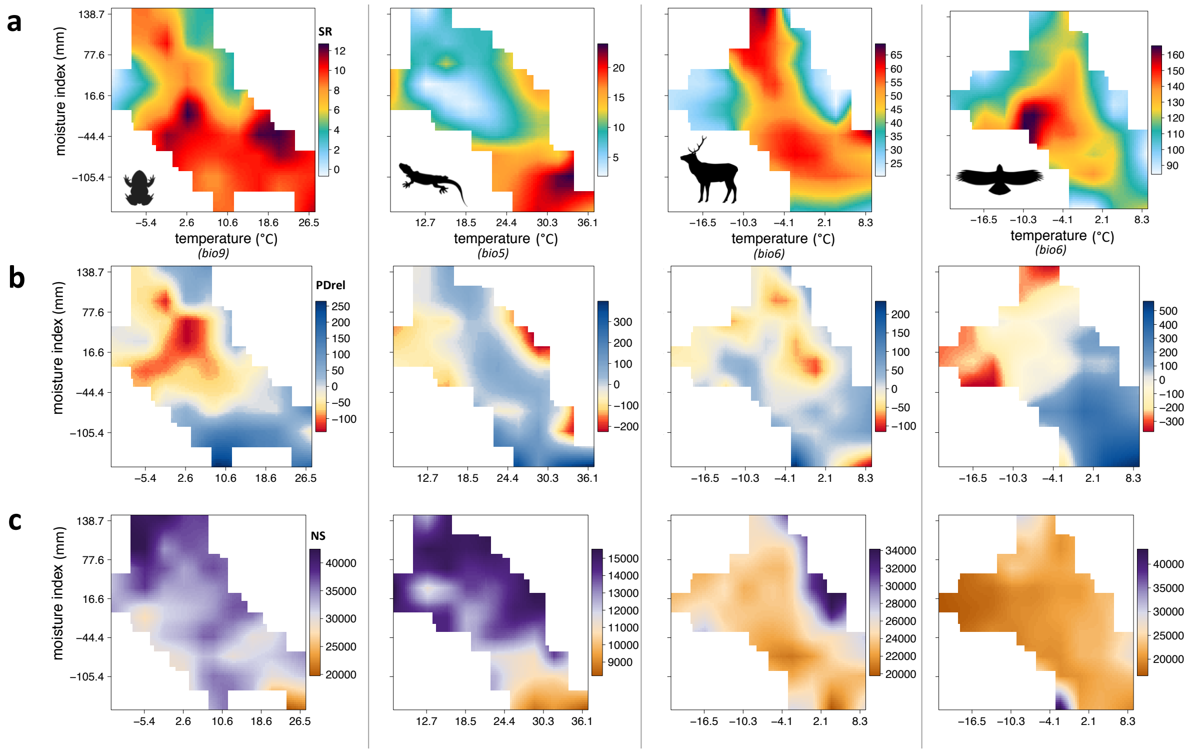

Supplementary Fig. 6. **Representation of mean niche size and diversity measures in environmental space**. **a**, Species richness (SR), **b**, relative phylogenetic diversity (PDrel), and **c**, mean niche size (NS) per pixel among all species for amphibians (1st column), squamates (2nd column), birds (3rd column), and mammals (4th column). Silhouette images were taken unchanged from phylopic.org, courtesy of Pedro de Siracusa (amphibians), Ghedo and T. Michael Keesey (squamates) both available under CC BY-SA 3.0 (creativecommons.org/licenses/by-sa/3.0/), Steven Traver (mammals) available under CC0 1.0 (creativecommons.org/publicdomain/zero/1.0), and Shyamal (birds) available under CC BY 3.0 (creativecommons.org/licenses/by/3.0/).

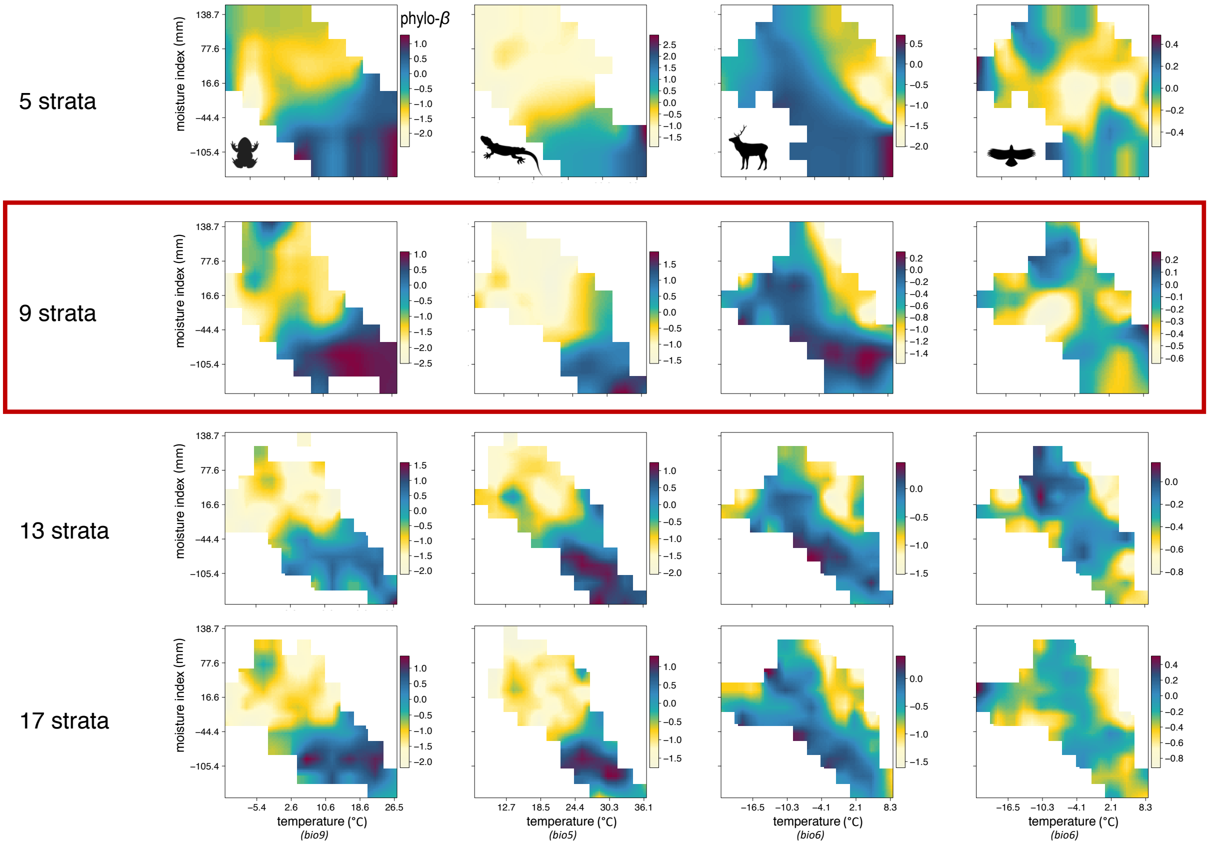

Supplementary Fig. 7. **Robustness of phylo-*ß* regarding the number of environmental sampling strata used.** True phylogenetic turnover (phylo-*ß*) is represented in environmental space (after removal of geographic distance effects) for amphibians (first column), squamates (second column), birds (third column), and mammals (fourth column) as a result of different numbers of environmental strata built prior to allocating the random sampling points to the different strata. The larger the number of strata, the smaller is the environmental distance between strata. Results within the red bar (9 strata) represent those used in the main text and are therefore identical to Fig. 2. Silhouette images were taken unchanged from phylopic.org, courtesy of Pedro de Siracusa (amphibians), Ghedo and T. Michael Keesey (squamates) both available under CC BY-SA 3.0 (creativecommons.org/licenses/by-sa/3.0/), Steven Traver (mammals) available under CC0 1.0 (creativecommons.org/publicdomain/zero/1.0), and Shyamal (birds) available under CC BY 3.0 (creativecommons.org/licenses/by/3.0/).

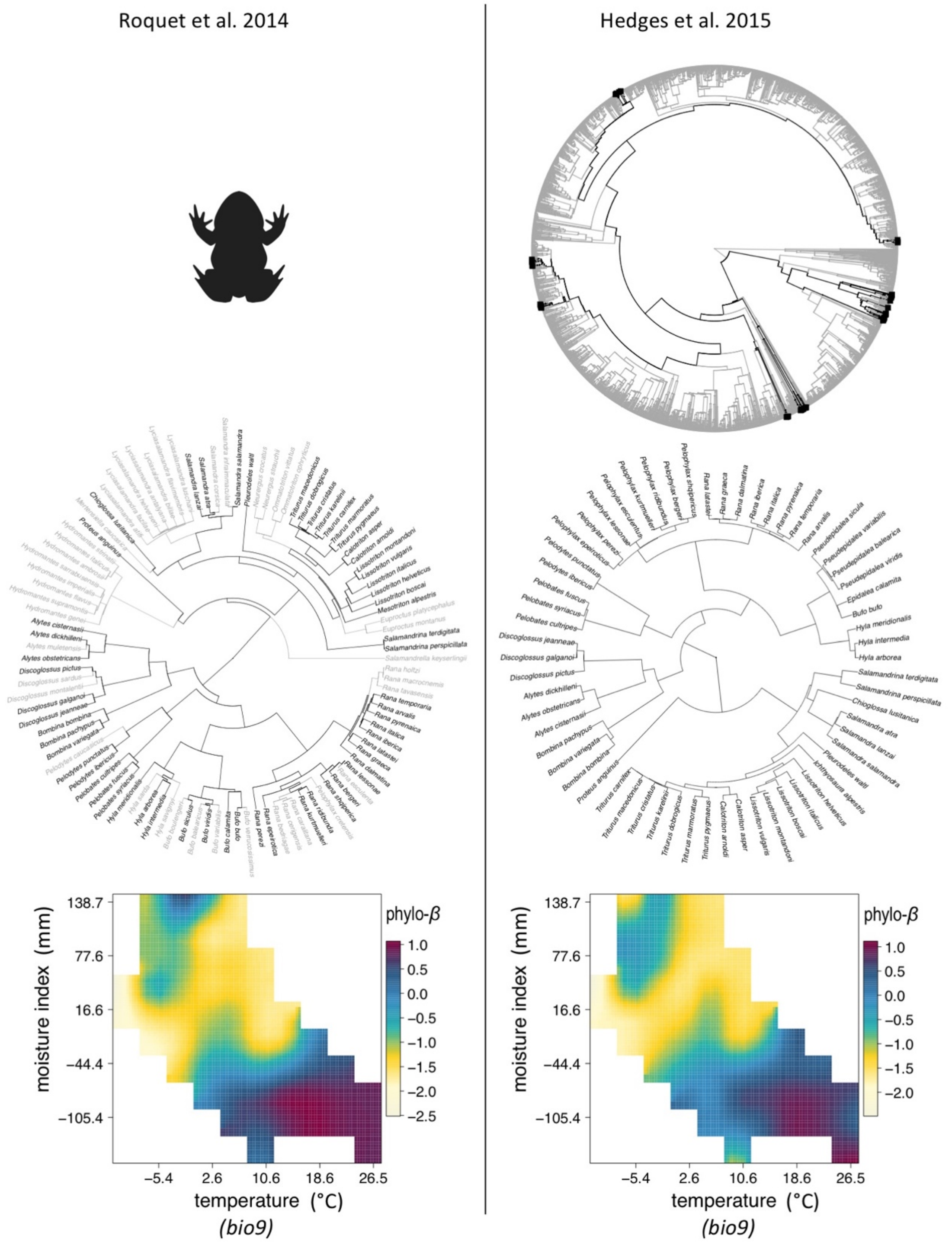

Supplementary Fig. 8. Robustness of phylo-*β* regarding the phylogenetic tree used for amphibians. Left column: phylogenetic tree from Roquet *et al.*[1] and associated phylo-*β* patterns derived thereof, below. Right column: phylogenetic tree from Hedges *et al.*[2] (Hedges, S. B., Marin, J., Suleski, M., Paymer, M. & Kumar, S., Tree of life reveals clock-like speciation and diversification, Mol. Biol. Evol., 2015, 32, 835-845, by permission of Oxford University Press) and associated phylo-*β* patterns derived thereof, below. Black tips and names represent the species included in this study, while grey tips and names represent species not occurring in our study area. The phylogeny at the top of the right column represents the global phylogenetic tree of amphibians from Hedges *et al.*[2]. The results to the bottom left represent those used in the main text and are therefore identical to the amphibian panel in Fig. 2. Silhouette image was taken unchanged from phylopic.org, courtesy of Pedro de Siracusa available under CC BY-SA 3.0 (creativecommons. org/licenses/by-sa/3.0/).

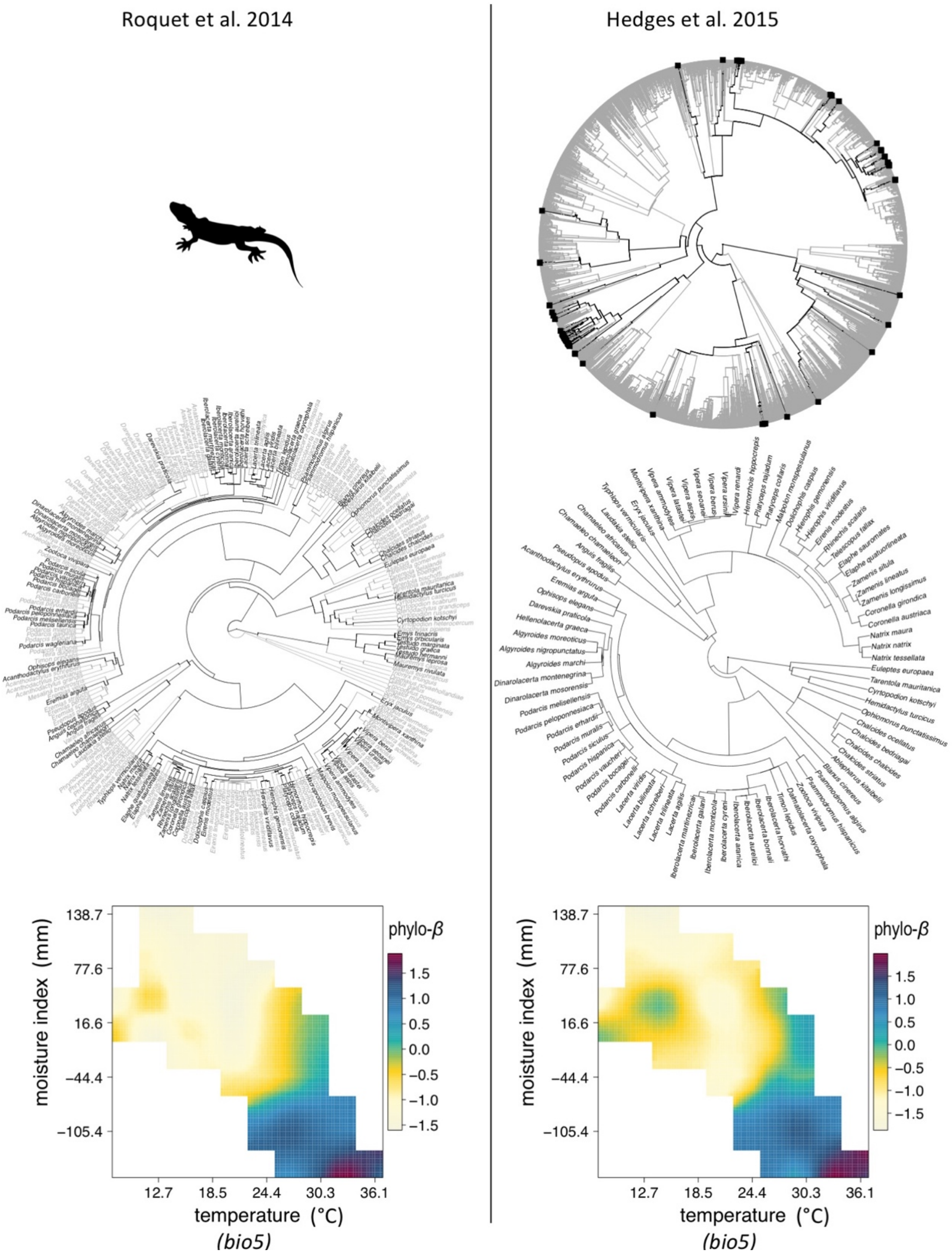

Supplementary Fig. 9. Robustness of phylo-*ß* regarding the phylogenetic tree used for squamates. Left column: phylogenetic tree from Roquet *et al.*[1] and associated phylo-*ß* patterns derived thereof, below. Right column: phylogenetic tree from Hedges *et al.*[2] (Hedges, S. B., Marin, J., Suleski, M., Paymer, M. & Kumar, S., Tree of life reveals clock-like speciation and diversification, Mol. Biol. Evol., 2015, 32, 835-845, by permission of Oxford University Press) and associated phylo-*ß* patterns derived thereof, below. Black tips and names represent the species included in this study, while grey tips and names represent species not occurring in our study area. The phylogeny at the top of the right column represents the global phylogenetic tree of squamates from Hedges *et al.*[2]. The results to the bottom left represent those used in the main text and are therefore identical to the squamate panel in Fig. 2. Silhouette image was taken unchanged from phylopic.org, courtesy of Ghedo and T. Michael Keesey available under CC BY-SA 3.0 (creativecommons.org/licenses/by-sa/3.0/).

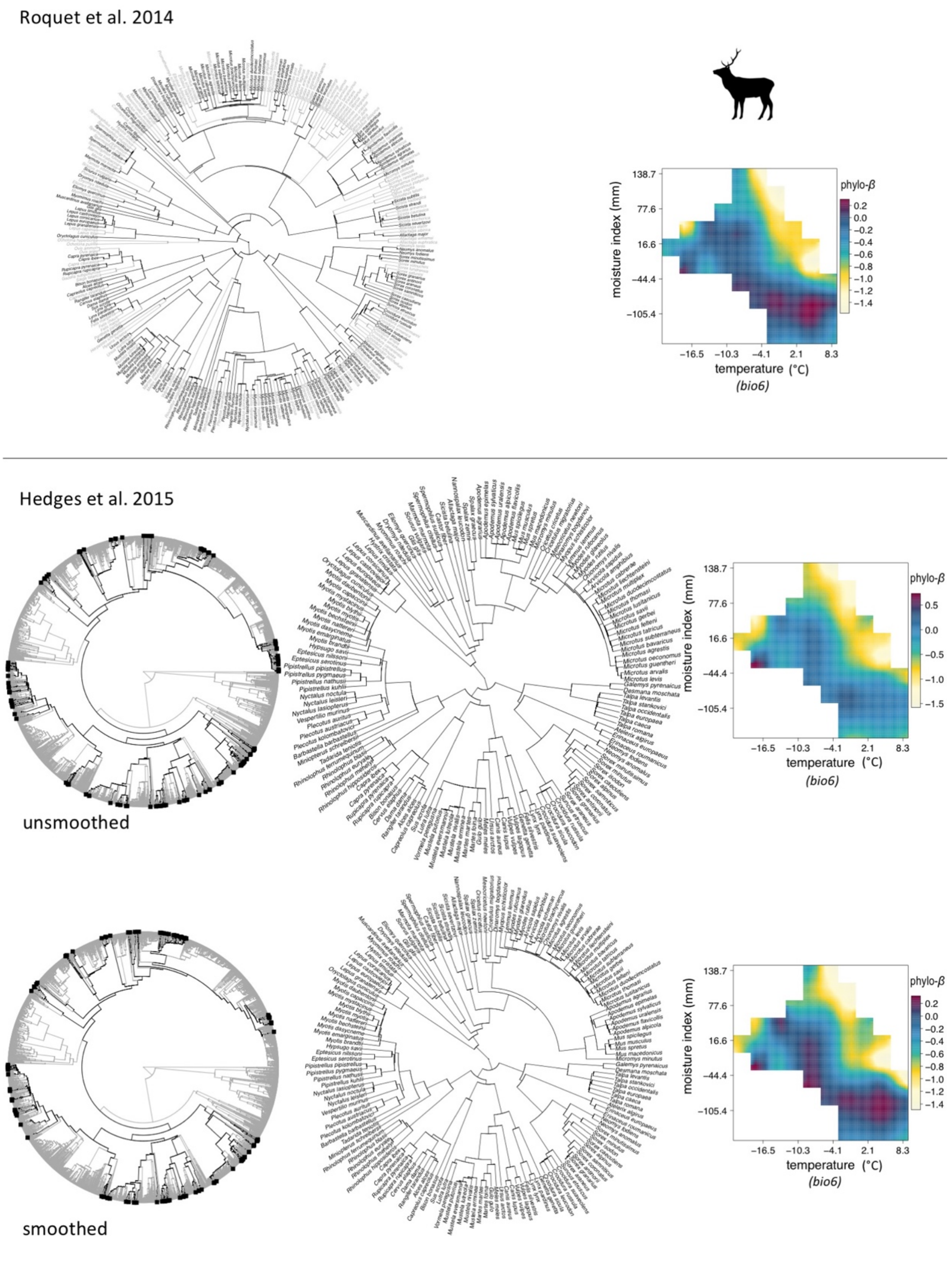

Supplementary Fig. 10. Robustness of phylo-*ß* regarding the phylogenetic tree used for mammals. Top row: phylogenetic tree from Roquet *et al.*[1] and associated phylo-*ß* patterns derived thereof, to the right. Middle and bottom row: phylogenetic tree from Hedges *et al.*[2] (Hedges, S. B., Marin, J., Suleski, M., Paymer, M. & Kumar, S., Tree of life reveals clock-like speciation and diversification, Mol. Biol. Evol., 2015, 32, 835-845, by permission of Oxford University Press) in the unsmoothed (middle) and smoothed (bottom) version and with the associated phylo-*ß* patterns derived thereof, to the right. Black tips and names represent the species included in this study, while grey tips and names represent species not occurring in our study area. The left-most phylogenies in the middle and bottom rows represent the global phylogenetic trees of mammals from Hedges *et al.*[2]. See methods for explanation of smoothed and unsmoothed versions of the phylogeny. The results to the top right represent those used in the main text and are therefore identical to the mammal panel in

Fig. 2. Silhouette image was taken unchanged from phylopic.org, courtesy of Steven Traver available under CC0 1.0 (creativecommons.org/publicdomain/zero/1.0).

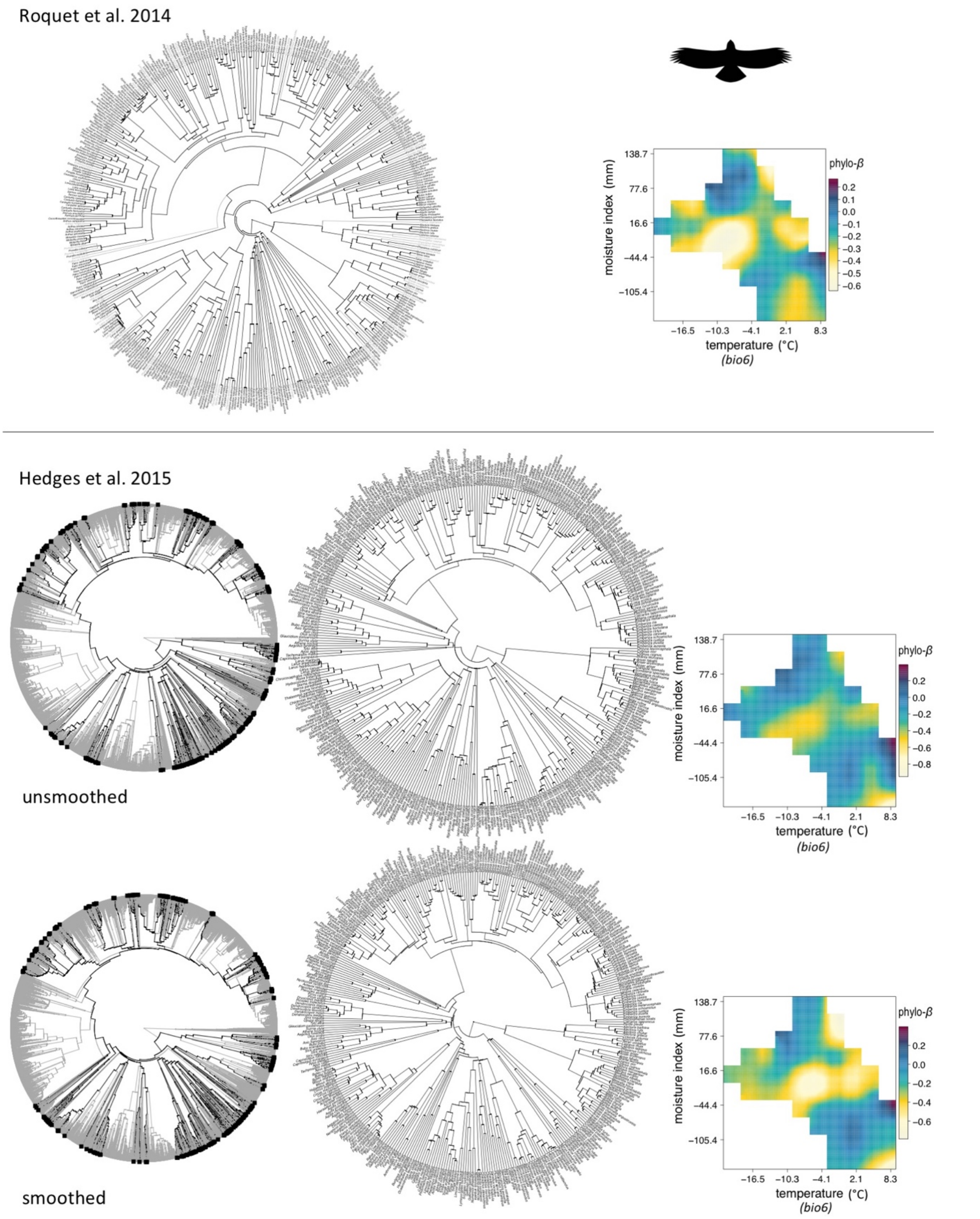

Supplementary Fig. 11. Robustness of phylo-*ß* regarding the phylogenetic tree used for birds. Top row: phylogenetic tree from Roquet *et al.*[1] and associated phylo-*ß* patterns derived thereof, to the right. Middle and bottom row: phylogenetic tree from Hedges *et al.*[2] (Hedges, S. B., Marin, J., Suleski, M., Paymer, M. & Kumar, S., Tree of life reveals clock-like speciation and diversification, Mol. Biol. Evol., 2015, 32, 835-845, by permission of Oxford University Press) in the unsmoothed (middle) and smoothed (bottom) version and with the associated phylo-*ß* patterns derived thereof, to the right. Black tips and names represent the species included in this study, while grey tips and names represent species not occurring in our study area. The left-most phylogenies in the middle and bottom rows represent the global phylogenetic trees of birds from Hedges *et al.*[2]. See methods for explanation of smoothed and

unsmoothed versions of the phylogeny. The results to the top right represent those used in the main text and are therefore identical to the bird panel in Fig. 2. Silhouette image was taken unchanged from phylopic.org, courtesy of Shyamal available under CC BY 3.0 (creative-commons.org/licenses/by/3.0/).

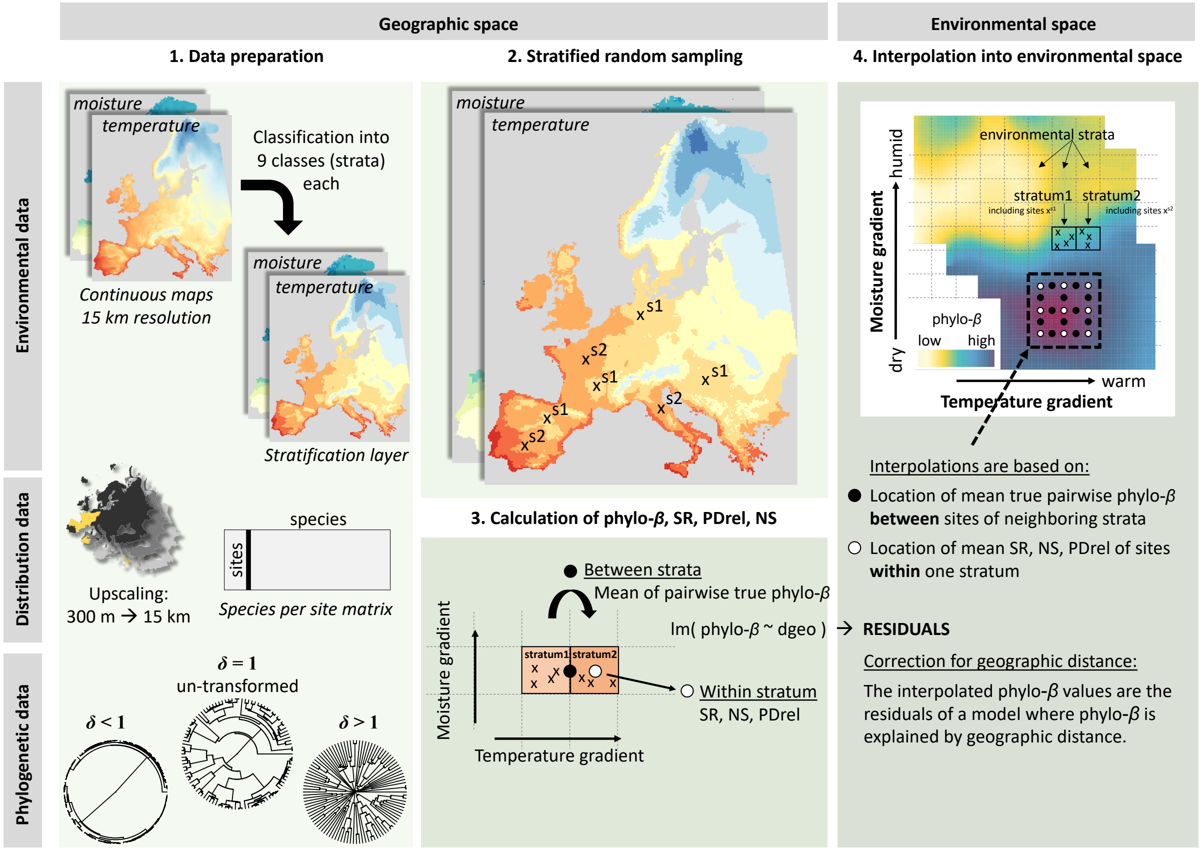

Supplementary Fig. 12. **Diagram illustrating the four main steps described in the method section of the manuscript.** Temperature and moisture data of Europe (continuous maps) were taken from Worldclim[3] and were stratified into 9 classes each (stratification layers). Sampling points were allocated geographically at random to sample the distribution data[4] within each stratum. Mean pairwise phylo-β (true turnover) was then calculated between sites of neighboring strata, after having removed the effect of geographic land distance from individual pairwise phylo-β measures. Phylo-β was calculated with phylogenetic trees that were untransformed or transformed by means of δ-transformation. The resulting mean pairwise phylo-β between strata was then mapped in environmental space by interpolation. See methods for more details in describing these four main steps.

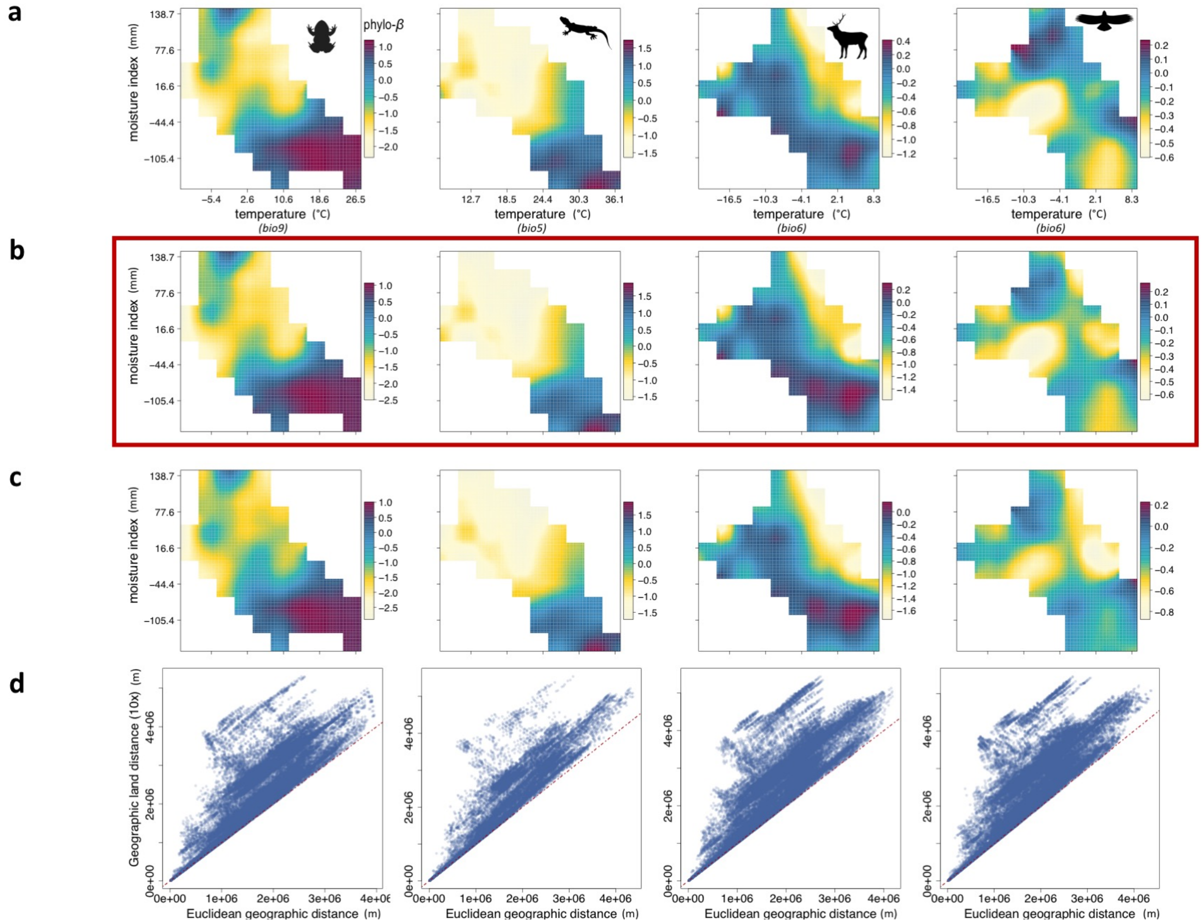

Supplementary Fig. 13. **Robustness of phylo-*β* regarding geographic distance removal.** True phylogenetic turnover (phylo-*β*) is represented in environmental space after removal of: Euclidean geographic distance effects (**a**), geographic land distance with 10x penalty for trajectory across sea pixels (**b**), geographic land distance with 100x penalty for sea trajectory (**c**) for amphibians (1st row), squamates (2nd row), birds (3rd row), and mammals (4th row). Results within the red bar (**b**) represent those used in the main text and are therefore identical to Fig. 2. **d** Scatterplot of Euclidean vs. geographic land (10x) distance, illustrating the larger distances obtained for many pairs of sample points. Silhouette images were taken unchanged from phylopic.org, courtesy of Pedro de Siracusa (amphibians), Ghedo and T. Michael Keesey (squamates) both available under CC BY-SA 3.0 (creativecommons.org/licenses/by-sa/3.0/), Steven Traver (mammals) available under CC0 1.0 (creativecommons.org/publicdomain/zero/1.0), and Shyamal (birds) available under CC BY 3.0 (creativecommons.org/licenses/by/3.0/).

Supplementary Table 1 Variance explained and correlation of climate variables with PCA axes 1 and 2

Bioclimatic variables	short	Axis 1	Axis 2
Proportion of variance explained		0.379	0.346
Cumulative proportion explained		0.379	0.725
Mean Diurnal Temperature Range	Bio2	0.263	-0.189
Temperature Seasonality	Bio4	0.137	0.386
Max Temperature of Warmest Month	Bio5	0.308	-0.269
Min Temperature of Coldest Month	Bio6	–	**-0.424**
Mean Temperature of Wettest Quarter	Bio8	0.2	0.209
Mean Temperature of Driest Quarter	Bio9	–	**-0.446**
Annual Precipitation Sum	Bio12	**-0.393**	-0.19
Precipitation Seasonality	Bio15	0.194	–
Precipitation of Wettest Quarter	Bio16	-0.345	-0.152
Precipitation of Driest Quarter	Bio17	**-0.392**	-0.125
Precipitation of Warmest Quarter	Bio18	-0.341	0.208
Precipitation of Coldest Quarter	Bio19	-0.273	-0.334
Moisture Index	MInd	-0.33	0.298

Six temperature and six moisture variables from the set of 19 bioclim variables from World-clim[3] in addition with the variable moisture index were used to generate two independent PCA axes. The first two rows indicate the proportion of variance explained and the cumulative proportion of variance explained by the first two axes. The correlation of each variable with the two axes are given below. The two variables with the highest correlation per axis are given in bold face.

Supplementary References

1 Roquet, C., Lavergne, S. & Thuiller, W. One tree to link them all: a phylogenetic dataset for the european Tetrapoda. *PLoS Curr.* **6**, doi:10.1371/currents.tol.5102670fff5102678aa5102675c5102918e5102678f5592790e5102648 (2014).

2 Hedges, S. B., Marin, J., Suleski, M., Paymer, M. & Kumar, S. Tree of life reveals clock-like speciation and diversification. *Mol. Biol. Evol.* **32**, 835-845 (2015).

3 Hijmans, R. J., Cameron, S. E., Parra, J. L., Jones, P. G. & Jarvis, A. Very high resolution interpolated climate surfaces for global land areas. *Int. J. Climatol.* **25**, 1965-1978 (2005).

4 Maiorano, L. *et al.* Threats from climate change to terrestrial vertebrate hotspots in Europe. *PLoS ONE* **8**, e74989 (2013).
